# *Perna canaliculus* and the Intestinal Microbiome

**DOI:** 10.3390/md15070207

**Published:** 2017-06-30

**Authors:** Emma Tali Saltzman, Michael Thomsen, Sean Hall, Luis Vitetta

**Affiliations:** 1 Sydney Medical School, The University of Sydney, Sydney 17200, Australia; esal4025@uni.sydney.edu.au (E.T.S.); mtho0952@uni.sydney.edu.au (M.T.); 2 Medlab Clinical, Sydney 17200, Australia; sean_hall@medlab.co

**Keywords:** bioactive, *Perna canaliculus*, green mussel, prebiotics, functional foods, intestinal microbiome

## Abstract

Natural medicines are often an attractive option for patients diagnosed with chronic conditions. Three main classes of bioactives that have been reported from marine mussel extracts include proteins, lipids and carbohydrates. Commercially, the most relevant species of marine mollusks belong to two genera, *Perna* and *Mytilus.* Specifically, the *Perna canaliculus* species has been repeatedly demonstrated to harbor anti-inflammatory compounds such as omega-3 polyunsaturated fatty acids (**ω**-3 PUFAs) that can ameliorate pro-inflammatory conditions, or proteins that can promote thrombin inhibitory activity. Recent clinical studies have posited that extracts from green-lipped mussels may lead to prebiotic activity in the intestinal microbiome that in turn has been reported to improve symptoms of osteoarthritis of the knee. Prebiotics have been reported to favorably interact with the intestinal microbiome through the proliferation of beneficial bacteria in the gut, suppressing exogenous and endogenous intestinal infections and promoting homeostasis by balancing local pro- and anti-inflammatory actions. Bioactive compounds from *Perna canaliculus* are functional foods and, in this regard, may positively interact with the intestinal microbiome and provide novel therapeutic solutions for intra-intestinal and extra-intestinal inflammatory conditions.

## 1. Introduction

The microbial diversity on this planet includes all bacteria, Archaea and most eukaryotic organisms [1,2]. Bacteria are omnipresent and the microbial diversity and abundance is very much determined by the biogeographical habitat (e.g., aquatic, terrestrial, atmospheric and living organisms) that is colonized. Bacterial activity can hence invariably affect the environment that is occupied [1].

**Figure 1:**
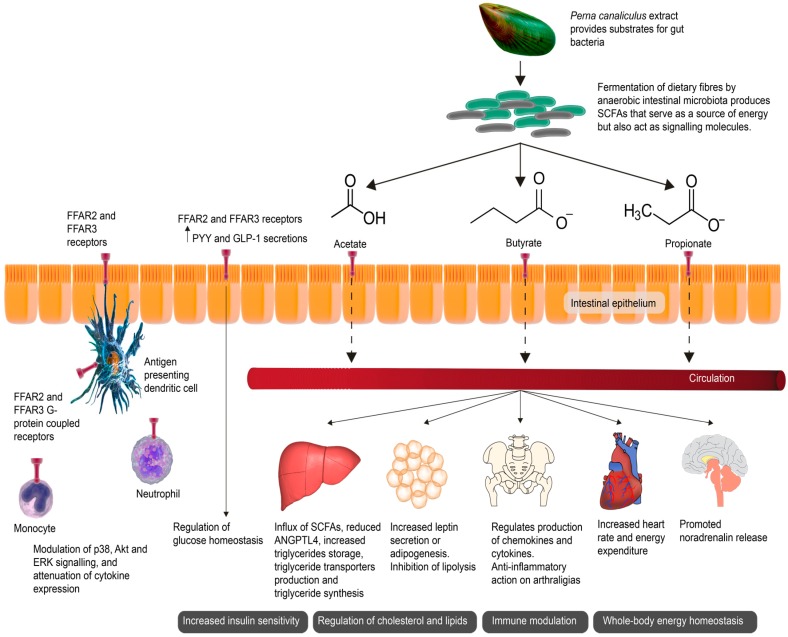
Short-chain fatty acids (SCFAs) as signaling molecules affected end-organ health. The green-lipped mussel (GLM) promotes anaerobic intestinal bacteria capable of producing SCFAs through the fermentation of dietary fibers. SCFAs serve as a source of energy for epithelial cells but also act as signaling molecules. SCFAs activate various receptors including the G-protein-coupled receptors Free Fatty Acid Receptors 2 and 3 (FFAR2 and FFAR3), expressed in the intestinal mucosa and on various immune cells. The activation of FFAR2 and FFAR3 stimulate the release of glucagon-like peptide-1 (GLP-1) and gut hormone peptide YY (PYY) in the intestine, improving insulin secretion. Hepatic uptake of SCFAs is associated with a decrease in angiopoietin-like 4 (ANGPTL4). ANGPTL4 regulates the uptake of triglycerides in adipocytes by inhibiting circulating lipoprotein lipase, thus promoting lipid clearance. The activation of FFAR2 and FFAR3 also promotes the release of noradrenalin, increasing the heart rate and energy expenditure, and further helping to maintain whole-body homeostasis [11,12].

In humans, immunological tolerance and the maturation of the intestinal mucosa has been reported to begin in utero [3,4]. The intestinal tract in humans harbors a complex set of bacterial taxa that has been intimately associated with phases of health and disease over a lifetime [5,6]. Symbiotically, the intestinal tract and the residing bacterial populations comprise one of, if not the most, metabolically and immunologically active organs in the human body [7]. Intestinal bacteria perform dual roles in an effort to maintain local homeostasis. Intestinal bacteria provide the impetus to ferment non-digestible dietary fibers to produce metabolites of short-chain fatty acids (SCFAs), which enables maximal energy extraction from dietary intake and fuels gut neuro-endocrine hormones, as well as regulating immunity. The definition of prebiotics has been derived from reports that suggest polysaccharides, including inulin, oligofructose (product of inulin), fructooligosaccharides (FOS) (synthetic polysaccharide of sucrose origin), and galactose- and xylose-containing oligosaccharides yield bifidogenic health effects in the presence of intestinal bacteria [8]. Therefore the current perception of prebiotics indeed refers to those compounds categorized as non-digestible food ingredients or substances (Figure 1), which pass undigested through the proximal part of the intestine (small bowel) and stimulate the growth and activity of health-promoting commensal bacteria that almost intuitively colonize the large bowel [9]. In addition to the bifidogenic effect the evidence from human studies reports that the impact of prebiotic supplementation on healthy physiological function is very much relevant to inducing satiety, increase breath-hydrogen excretion, and modulate intestinal peptides involved in appetite regulation [10].

Bacteria residing in the intestinal tract have a potent ability to ferment otherwise indigestible complex dietary fibers into short-chain fatty acids (SCFAs) [13]. Clinical and experimental data has supported the benefits of SCFA production, with studies reporting that the gut microbiota of healthy volunteers differs from subjects with chronic diseases or infections and that significant depletions in bacterial species associated with SCFA production correlates with increases in opportunistic pathogens [14]. Whilst the metabolism of *P. canaliculus* by commensal bacteria is yet to be explored extensively, in vitro data has demonstrated their capacity to ferment and metabolize a common anti-arthritic medication, d-glucosamine [15,16,17]. By modifying the bioactive components of food, the intestinal microbiota can influence the exposure to nutrients that have health or disease modifying potential [17]. *P. canaliculus* has been demonstrated to indirectly target the intestinal microbiota and in part influence intestinal bacterial biodiversity, growth and metabolic activity [17].

## 2. Intestinal Bacterial Cohort

The genera of bacteria that are consistently reported as key for producing and maintaining health-promoting activities in the prebiotic literature are largely limited to the *Lactobacillus* and *Bifidobacterium* genera. However, other bacterial groups that were once considered responsible for producing unfavorable health outcomes in the intestines, such as *Bacteroides* and *Clostridia*, have been shown to be associated with beneficial physiological effects following extensive research with prebiotics. These findings have led an enhanced appreciation of the capacity of the intestinal microbial cohort as a significant therapeutic target in various pathophysiological contexts [14,18,19].

Harboring a biodiverse abundance of bacteria, the intestinal microbiota is critical in shaping host health (32). Functioning as a protective barrier against pathogen translocation and preventing overgrowth of opportunistic bacteria, the intestinal microbiome can trigger deleterious host health effects as a consequence of dysbiosis [20]. The intestinal microbial cohort is very much dependent on the provision of viable substrates so that the bacteria can generate an array of metabolites such as vitamins and organic acids (i.e., branched-chain fatty acids and short-chain fatty acids) that can beneficially impact local and end-organ sites by maintaining homeostasis [21]. Homeostatic balance, characterized by a diverse and balanced microbiota, can be disrupted in the intestines, inducing pathological imbalances that are increasingly linked to the progression of metabolic disorders such as obesity and metabolic syndrome [20]; immune-meditated pathologies such as *Clostridium difficile* and *Campylobacter jejuni* infections; large bowel and liver cancers; inflammatory bowel diseases; cardiovascular disease; malnutrition; chronic kidney disease; autoimmune arthritis; multiple sclerosis, Parkinson’s disease; mood disorders; and food/environmental allergies.

Dysbiosis, a gut barrier abnormality of the intestinal epithelia, may also play a central role in nutraceutical efficacy to help ameliorate these conditions. Modulation of the intestinal microbiota is a potential novel therapeutic modality to improve the health outcomes of such diseases and conditions. Immunomodulation, by which an immune response is modified in either a positive or negative manner through the administration of a pharmacological agent or other compound that could be classed as a prebiotic, is a quality that can be harnessed from various naturally occurring marine invertebrates such as the green-lipped mussel [22].

## 3. Bioactive Molecules and Compounds from Green-Lipped Mussel

The species of *Perna canaliculus* is a member of the *Bivalvia* class from the *Mollusca* phylum within the *Mytilidae* family. Paleontological data that dates back to 60 million years ago to the Eocene period documents the existence of the *Perna* genus [23]. Containing both green and brown mussel species, the genus is located primarily in the marina of the Southern Hemisphere, but have also been discovered on the northern coastal regions of South America and North Africa [23]. There are three species within the *Perna* genus that are dispersed across the globe: the *P. viridis* species (Asian green mussel) in Indo-Pacific region; the *P. perna* (brown or rock mussel) species from Atlantic regions, and; the *P. canaliculus* species, limited to the marine regions of New Zealand, which has been sustainably farmed for commercial purposes since the early 1970s [23]. A number of bioactive compounds from the *Mytilus* and *Perna* genuses are found within the *P. canaliculus* species extract, which is emerging as the most carefully and intensely investigated compound for medicinal and therapeutic effects. *P. canaliculus* has been utilized in the design of commercial therapeutic agents of arthralgia in humans and animals alike. Current studies investigating the therapeutic efficacy of *P. canaliculus* have only produced low-level evidence, however, an indication remains that green-lipped mussels (GLMs) have therapeutic potential as adjunctive agents for rheumatoid arthritis (RA) [22], osteoarthritis (OA) [24,25] asthma [26] and intestinal complaints [27]. 

Clinical and experimental data has shown various mussel species possess anti-oxidant, anti-hypertensive, anti-bacterial, anti-thrombin and anti-coagulant properties derived from their proteins, peptides and amino-acid constituents.

Pernin remains the only bioactive protein that has been found in the cell-free hemolymph of the *P. canaliculus* extract. Composed of close to 500 amino acids, pernin is an aggregating, non-pigmented, glycosylated protein extract. With a particularly high concentration of histidine and aspartic acid residues, pernin is a serine protease inhibitor. However, pernin only shows a weak level of anti-thrombin activity [28]. The homogenized meat of a whole mussel contains approximately 0.2 mg of pernin per mussel [28]. Owing to the high proportion of pernin in the hemolymph, it has been suggested that the protein must therefore be responsible for important functions in the mussel [28]. Pernin may represent an otherwise unclassified category of bivalve proteins with a distinctive structure, origin and function [28]. *M. edulis* has also been reported to contain another anti-coagulant peptide [29]. *P. canaliculus* supplementation has not been associated with significant side effects or allergies following its use and administration in numerous clinical studies [30]. An extremely rare adverse effect of non-granulomatous cholestatic hepatitis was associated with *P. canaliculus* extract consumption [30], with one of the two patients who reported this complication having a pre-existing hepatic disorder [31].

### 3.1. Mechanism of Intestinal Actions

The anti-inflammatory effect of *Perna* has been attributed to a number of factors, one of which being its ability to reduce the biosynthesis of pro-inflammatory prostaglandins [22]. It has been suggested that *P. canaliculus* contains active inhibitors of prostaglandins, as demonstrated by an increase in the gestation period of rats following the administration of *P. canaliculus* extract powder [32]. Clinical studies have yet to be completed assessing the therapeutic efficacy of mussel proteins and peptide fractions in humans. Reviews have suggested that the intestinal microbiota may be involved in the down-regulation of intestinal mucosal inflammation and, as such, the administration of compounds like *P. canaliculus* to manage symptoms of inflammatory conditions like OA and potentially RA are viable adjunctive therapies [33]. 

### 3.2. Immunomodulation

Early studies with *murinae* models have shown that the lipid-rich fraction of the GLM exhibits synergistic anti-inflammatory properties that are characteristic of immunomodulation when administered in combination with oral non-steroidal anti-inflammatory drugs (NSAIDs) and analgesic medications including prednisone, pentoxifylline or meloxicam [34,35]. Following tandem delivery, paw swelling was significantly reduced in rats with adjuvant-induced arthritis and zymosan-induced paw inflammation [35]. In fact, the symptom relief from tandem therapies was more effective than that of NSAID therapy alone [35]. *P. canaliculus* extract powder has shown synergistic and anti-inflammatory activity when administered in combination with other NSAIDs. Experimental data has demonstrated that the stabilized whole extract powder of *P. canaliculus* decreases the severity and episodes of intestinal barrier damage in rat models [33,34]. The anti-inflammatory activity of acetylsalicyclic acid and indomethacin is further supported by reports that lipid and whole extract powders affect gastroprotective effects [36]. 

Experimental evidence from an animal model of inflammatory bowel disease (IBD) has suggested that the lipid-rich fraction of the supercritical extract of *P. canaliculus* may exert gastroprotective effects to treat colonic damage [27], as results reported improvement in intestinal inflammation markers and intestinal morphology in chemotherapy-induced mucositis in an animal models [27]. Studies conducted recently in humans reported that the administration of a whole *P. canaliculus* extract may support intestinal function and potentially protect the intestinal tract when delivered in conjunction with analgesic and anti-inflammatory medications in OA patients [17,25].

Approximately 70% of an extract of whole mussel *P. canaliculus* is protein. Exhibiting both humoral and cellular immunemodulating effects, previous investigations have demonstrated the strong anti-inflammatory profile of *Perna* [22]. Furthermore, the anti-inflammatory potential of *Perna* has been shown to be as effective as NSAIDs in its ability to reduce inflammation in murine models of carrageenan-induced footpad edema [22]. Animal and in vitro model data indicates that fractionated extracts of whole extract powder derive their anti-inflammatory and immunomodulating properties from an active agent that is predominantly a protein moiety or an actual protein macromolecule [37,38]. Unfortunately, high-level evidence for the bioactive high molecular weight protein is lacking and requires further analysis [37,38]. Furthermore, a recent study suggests that marine compounds such as the red seaweed *Chondrus crispus* presents evidence that when orally administered may act as a prebiotic [39]. The prebiotic effects suggested were consistent with influencing the composition of the intestinal microbial communities, improvement of intestinal health and immune modulation in the animals that were supplemented with *Chondrus crispus*. 

The mechanism of anti-inflammatory activity and immunomodulating properties of *P. canaliculus* has been hypothesized to be a result of the inhibition of the production of pro-inflammatory cytokines [22]. Blocking arachidonic metabolism through cyclooxygenase (COX) and lipoxygenase pathways, *P. canaliculus* can efficiently block the action of COX-1 and COX-2 enzymes via a dose-dependent mechanism. It is this blockage in COX pathways that is linked with reduced levels of multiple pro-inflammatory cytokines, including interleukin (IL) 1, IL-2, Il-6 and tumor necrosis factor alpha (TNF-alpha) [22]. Regulating the pro-inflammatory network, TNF-alpha plays a disease-promoting role in RA. Moreover, *Perna* is perhaps a safer therapeutic modality than NSAIDs in light of the preferential blockage of the pro-inflammatory COX-2 enzyme over the physiologically superior COX-1 enzyme. With numerous inflammatory and autoimmune responses triggered by abnormally high levels of systemic TNF-alpha, compromising or disrupting their production with the use of naturally occurring organisms or compounds such as *M. edulis* and *P. canaliculus* is a viable approach [22]. Lyprinol contains the oil of *P. canaliculus* and displays anti-inflammatory effects in vivo and in vitro [40]. Table 1 details randomized clinical trials that have investigated the effect of *P. canaliculus* supplementation in the treatment of arthritic diseases.

### 3.3. Anti-Microbial Activities

Anti-microbial peptides (AMPs) have been detected in the hemolymph of mussels and form the basis of their innate immunodefense system. Providing protection against bacterial, fungal and viral invasions, these mussel-origin AMPs are of particular interest and specifically those derived from the Blue mussel (*Mytilus edulis*) and the Mediterranean (or Blue) mussel (*Mytilis galloprovincialis*) species. The *M. edulis* species has been shown to possess numerous cysteine-rich peptides that impart potent bactericidal (i.e., against Gram-positive organisms, e.g., *Enterococcus faecalis*, *Staphylococcus aureus* and Gram-negative bacteria, e.g., *Escherichia coli* bacteria) and anti-fungal (i.e., *Neurospora crassa* and *Fusarium culmorum*) functionality [41]. The AMPs identified have been classified as isoforms of the peptide families of defensins, mytimycin and mytilin with big defensins [38,41]. The crustacean hemolymph, specifically of crab origin, possesses a range of AMPs that act as endogenous antibiotics, as well as participating in pro-inflammatory activity, wound repair and regulating adaptive immunity responses [33]. These findings of the potency of whole mussel extract powder has provided the impetus for integrating marine peptides into novel pharmaceutical developments [42].

### 3.4. Cardiovascular Effects 

The fermented *M. edulis* possesses angiotensin I converting enzyme (ACE) inhibiting peptides that exert anti-hypertensive effects, as reported by in vivo rat model data [43]. 

Conversely, the lipid fraction of *Perna* has been extensively investigated. Associated with potent anti-inflammatory activity, *M. edulis* and *P. canaliculus*, like other marine organisms, are naturally low in dietary cholesterol [44]. However, it is their possession of cholesterol-lowering phytosterols and **ω**-3 PUFAs that separate these marine organisms from the naturally omega-6 (**ω**-6) rich terrestrial organisms [45] and impart therapeutic properties. The **ω**-3 PUFA portion has been suggested to be responsible for helping to reduce risk factors associated with cardiovascular disease, as well as helping to relieve symptoms of inflammatory disorders like OA [44].

## 4. The Microbiome and Omega-3 Fatty Acids

### 4.1. Changes in the Intestinal Microbiome

Changes in the microbiota profiles and gut functionality were observed in patients diagnosed with OA and subsequently treated with a *Perna canaliculus* meat freeze-dried powder. The most significant change was a decrease in the *Clostridia* spp. This study suggests that the mussel meat powder may serve to control metabolic and immunity activity of the intestinal microbiome. *Clostridia*, previously established as a potent modulator of Th17 and CD4+ regulatory cells, correlates with inflammation and Gastrointestinal Symptom Rating Scale (GSRS) scores and OA symptoms [17]. 

Several animal and human studies have shown that a diet rich in **ω**-3 PUFAs strongly alters the microbiome composition, although one study found that while **ω**-3 PUFAs had a significant effect on insulin sensitivity and hsCRP, they had no effect on gut microbiota in a study of 60 overweight adults [55]. A diet rich in the fish fatty acids EPA and DHA for 15 days decreased *Helicobacter*, *Clostridiales* bacterium, *Sphingomonadales* bacterium and *Pseudomonas* species *Firmicutes* in mice [56], while a diet rich in sardines for six months (100 g sardines providing 3 g EPA and DHA) was shown in 35 drug-naïve patients with type 2 diabetes to be associated with a shift in the intestinal microbiome. Both the fish and the standard diabetes diets decreased phylum *Firmicutes* (*p* = 0.04) and increased *E. coli* concentrations (*p* = 0.01) compared to baseline. However, only the fish diet was associated with a decreased in the ratios of *Firmicutes* to *Bacteroidetes* (*p* = 0.04) and an increase in the ratio of *Bacteroides* to *Prevotella* (*p* = 0.04) compared to baseline [57].

The ratio of pro-inflammatory **ω**-6 to anti-inflammatory **ω**-3 PUFAs is also important for gut health. High intakes of dietary fat, including palm oil, olive oil, safflower oil or linseed/fish oil for 16 weeks was shown by high-throughput 16S rRNA sequencing to significantly alter the microbiome of C57BL/6J mice. The linseed/fish oil diet triggered significant growth in the genus level of *Bifidobacterium* compared to mice fed a low-fat high-maize starch diet (*p* < 0.05) [58]. Intestinal dysbiosis and low-grade systemic inflammation was reported to have been induced in the group of mice that were allocated to and fed a diet high in **ω**-6 PUFAs. Mice fed a diet supplemented with **ω**-3 PUFAs showed changes in their intestinal bacterial profile composition, which led to a decrease in lipopolysaccharide (LPS) and therefore enhanced gut barrier integrity. This chain of events ultimately reduced levels of metabolic endotoxemia and inflammatory activity [59]. A diet high in **ω**-6 PUFAs from corn oil was further shown in a rodent model of colitis to increase the level of *Enterobacteriaceae*, *Segmented Filamentous Bacteria* and *Clostridia* spp. which aggravated and disturbed the intestinal barrier, immune cell proliferation, the expression of prostaglandin E2 and the passage of *C. rodentium* across the intestinal mucosae. Adding **ω**-3 PUFAs reversed these inflammatory-inducing bacteria and enriched the level of *Lactobacillus* and *Bifidobacterium* species [60].

### 4.2. SCFAs as Signaling Molecules for End-Organ Health

Fermentation of dietary fibers by anaerobic intestinal microbiota produces short-chain fatty acids (SCFAs). Evidence from aforementioned studies of **ω**-3 PUFAs from a variety of sources, including fish and *P. canaliculus,* suggest that certain oils may have a beneficial effect on the microbiome by triggering the growth of certain intestinal bacterial species, which may contribute to the microbiome’s ability to produce SCFAs. Primarily serving as an energy source, SCFAs also act as signaling molecules (Figure 1) [17]. Clinical data from OA patients treated with a *P. canaliculus* containing GLM extract reported significant improvement in GSRS scores, supporting the hypothesis that the metabolism of *P. canaliculus* containing GLM compounds may generate the production of SCFA metabolites. This hypothesis provides a plausible mechanism of action of *P. canaliculus* as a therapeutic nutraceutical [17]. Further studies have reported a gastroprotective effect of GLM, with intestinal dysfunction significantly improved in GLM-treated subjects further suggesting that *P. canaliculus* may play an important role in intestinal sequelae and intestinal barrier maintenance [25]. From the rudimentary analysis of fecal samples by Coulson et al. [17], the study showed that *Bacteroides* (a genus of Gram-negative, obligate anaerobic bacteria) as a group were increased at the end of the *P. canaliculus* supplementation period, indicating that *P. canaliculus* possibly increased SCFA-producing intestinal bacteria. The majority of SCFAs produced are taken up in the colon with only 5–10% secreted via the feces. Only a small percentage is absorbed by passive transfusion, with the majority absorbed by monocarboxylate transporters (MCT), SLC5A8 (sodium coupled transporters (SMCT)), as well as the G-protein-coupled receptors Free Fatty Acid Receptors 2 and 3 (FFAR2 and FFAR3). The SCFAs of acetate and propionate have been found to primarily activate FFAR2, whereas propionate and butyrate are responsible for the activation of FFAR3. A range of immune cells have been shown to express FFAR2 and exert immunomodulating effects. The activation of FFAR2 and FFAR3 stimulates glucagon-like peptide-1 (GLP-1) secretion and gut hormone peptide YY (PYY) in the intestine, resulting in improved insulin release. SCFAs produced by fermentation of dietary fibres by a healthy microbiota thus help manage blood glucose levels. Lack of dietary fibre is conversely related to an increased risk of developing insulin resistance and eventual onset of type 2 diabetes mellitus. FFAR2 and FFAR3 also helps maintain whole-body energy homeostasis by regulating leptin, adipogenesis, fat accumulation and lipolysis in adipose tissue as well as regulating hepatic cholesterol synthesis and triglyceride storage. Hepatic uptake of SCFAs is also associated with a decrease in angiopoietin-like 4 (ANGPTL4). ANGPTL4 regulates the uptake of triglycerides in adipocytes by inhibiting circulating lipoprotein lipase thus promoting lipid clearance. Activation of FFAR2 and FFAR3 also promote the release of noradrenalin, increasing heart rate and energy expenditure, and further helping to maintain whole-body homeostasis [11,12].

### 4.3. Gut Synthesis of Butyrate

A majority of the butyrate produced in the intestinal tract is posited to originate from carbohydrates. However, a subset of specialized intestinal bacteria has been identified that facilitates the conversion of lactate and acetate to butyrate. *Intestinimonas* strain AF211, a commensal bacterium, has been shown to convert lysine into butyrate and acetate, suggesting that proteins have the capacity to provide a source of butyrate in the colon [61]. However, only 5% of gut SCFAs are derived from lysine conversion [62]. SCFAs in the gut are in a state of flux. Numerous bacterial species are involved in the production and interconversion of SCFAs. A large group including *Bacteroides*, *Proteobacteria*, *Bifidobacteria*, *Clostridia*, and *Eubacteria* produce acetate. *E. rectale* and *Roseburia* spp. can utilize actetate to produce butyrate. Butyrate can also be converted back to actatate by sulfate- or nitrate-reducing acetogenic bacteria [63].

Marine lipids may also influence the microbiome and end organs by enhancing butyrate-producing bacteria. Animals with early life stress, induced by maternal separation, have been shown to develop dysbiosis characterized by a decreased ratio of *Bacteroidetes* to *Firmicutes* and the induction of inflammatory cytokines in plasma. Long-term administration of EPA and DHA reversed this shift. Maternal separation induced *Akkermansia* spp., a bacteria associated with degradation of mucus, and microbial translocation across the mucosa, exacerbating gut inflammation [64]. *Akkermansia* spp. is also a butyrate-producing bacterium. A diet containing 600 mg **ω**-3 PUFAs daily for 14 days has been shown in a 45-year-old male to increase butyrate-producing bacteria *Roseburia intestinalis* and *Eubacterium rectale*, which became the predominant species. The study found that **ω**-3 PUFAs for two weeks were associated with increases in *Eubacterium*, *Roseburia*, *Anaerostipes*, *Coprococcus*, *Subdoligranulum*, *and Pseudobutyrivibrio*, which are genera associated with butyrate production. These changes were reversed after a 14-day washout period [65].

## 5. Discussion

Nutraceutical supplements, such as GLMs and glucosamine sulfate (GS), when administered orally, are known to be metabolized by the intestinal microbiota [7,33]. While the metabolism of GLM extract is yet to be investigated comprehensively, preliminary in vitro studies have confirmed that GS extract is fermented and metabolized by commensal intestinal bacteria [15,16,66].

Understanding the distribution of the intestinal microbiome can help further progress effective adjunctive treatments that can abrogate unwanted side effects of medical therapies. Mucositis and **ω**-3 PUFAs is such an example. Fish fatty acids such as EPA and DHA from a lipid-rich extract of *P. canaliculus* and a combination of the two fatty acids have been investigated in animal models of 5-FU-induced mucositis. DHA was reported as more beneficial than EPA with the combination having the best effects. Fish oils and a lipid-rich extract from *P. canaliculus* have also been shown to ameliorate the toxic effects of 5-FU on the intestines. Pretreatment with DHA in conjunction with protein supplementation has been shown in rats to reduce apoptosis of intestinal cells following 5-FU treatment [67]. **ω**-3 PUFAs may enhance the efficacy while reducing adverse effects of cytotoxic drugs. 5-FU in combination with fish oils has been shown to increase the survival rate in carcinogen-treated animals. Synergism of 5-FU and fish oil was also reflected in significant inhibition in tumor growth in a model of colon cancer. Fish oils also ameliorated 5-FU-induced toxicity as substantiated by a marked improvement in the structural and functional alterations of various organs [68]. In a study comparing Lyprinol with fish oil and olive oil, pretreatment with **ω**-3 PUFAs from both fish oils and *P. canaliculus* (Lyprinol) and olive oil for five days decreased 5-FU-induced mucositis in rats. The weight of the small intestine was significantly greater in animals receiving fish oils from and *P. canaliculus* compared to controls (*p* < 0.05). Lyprinol was also associated with less severe histological damage with the intestines having longer crypts and increased proliferation in the midsection of the small intestine compared to controls (*p* < 0.05), although it was unable to prevent 5-FU-induced weight loss, decreased feed intake and the weight of thymus and spleen [69]. The synergistic effect of DHA and EPA was also demonstrated in a mouse study where pretreatment was shown to reduce weight loss and preserve intestinal mucosa and integrity following 5-FU injection. The diet was associated with longer villi compared to controls. The diet also reduced inflammatory cytokine activation and expression, and upregulated anti-apoptotic genes [70].

Clinical and experimental data both suggest that **ω**-3 PUFAs may trigger a beneficial effect in metabolic syndrome-related conditions. In response to a diet enriched with **ω**-3 PUFAs, the intestinal microbiota is characterized by a shift in the composition and abundance of butyrate-producing bacterial species [65]. Such a shift in the microbial configuration provides a possible explanation as to how **ω**-3 PUFA supplementation ameliorates acute and chronic diseases associated with intestinal epithelial dysbiosis. With intestinal epithelial dysbiosis identified as a trigger for metabolic syndrome and the associated complications that ensue, the intestinal microbiota presents as a major target in the management and possible treatment of such pathologies. As the hepatic expression of the metabolic syndrome, non-alcoholic fatty liver disease (NAFLD), may present as another therapeutic target for marine-derived **ω**-3 PUFA supplementation [71]. Studies have shown that various supplements rich in **ω**-3 PUFAs reversed hepatic lipid accumulation and inflammatory markers in subjects with pre-existing hepatic steatosis. The results of such studies are underpinned by the hypothesis that **ω**-3 PUFAs alter the intestinal microbiome while concurrently regulating hepatic lipid metabolism [72]. Although data implicates configurational changes in the intestinal microbiome profile in the manifestation of chronic and acute adverse health effects, a requisite for prospective studies remains to establish definitive mechanisms of actions and pathogenesis.

## 6. Conclusions

Understanding how the bioactive compounds derived from *P. canaliculus* interact and communicate with the microbiome populating the intestinal tract may help to facilitate and guide the design of novel therapeutic agents to manage intra-intestinal and extra-intestinal inflammations [17]. An altered intestinal microbial profile is purported to be a significant contributor to the therapeutic efficacy of GLMs and other nutraceutical supplements [17]. Intestinal epithelial dysbiosis is implicated in the onset and maintenance of acute and chronic diseases, with **ω**-3 PUFA rich *P. canaliculus* hypothesized to proliferate the growth of microbial species linked with SCFA-production. This shift in the intestinal microbial composition and increased production of SCFA, particularly butyrate, is associated with anti-inflammatory, anti-microbial and immunomodulatory effects. As such, in vitro and RCTs have shown *P. canaliculus* and other marine-derived omega-3 fatty acid rich supplements as potential therapeutic agents for arthritic diseases, metabolic syndrome and associated complications, as well as chemotherapy-induced mucositis. However, it is important to note that the various RCTs that have been conducted to date using commercial preparations containing *P. canaliculus* vary greatly [73] and, as such, further investigations into the active substance composition would be beneficial to confirm their physiological effects.

## Figures and Tables

**Table 1:** Clinical trials with *Perna canaliculus* extract for the treatment of arthritic diseases.

Study Type (*n*)	Intervention/Control	Results
RCT (*n* = 38) [46]	Intervention group: Mussel extract 1050 mg/day for 3 months Control received placebo for treatment period Both then received extract for further 3 months	Intervention group reported 31% responders vs. 14% in placebo NSD in responder numbers
RCT (*n* = 53) [47]	Intervention group: 6 Seatone capsules daily (dosage unknown) Placebo group: 6 placebo capsules 6-month duration	6 months: significant improvement in pain, function score and GA. Seatone significantly superior to placebo in 4 assessed criteria
RCT (*n* = 30) [48]	Group A: lipid extract–3 capsules/day (210 mg) Group B: stabilized mussel powder–5 capsules/day (1150 mg) Treatment period (3 months) then both groups given lipid extract for further 3 months	End of treatment period: significant improvements for both groups for AI, VAS pain, FI and patient and GA NSD found between treatment groups
RCT (*n* = 80) [49]	Lyprinol^®^ (dose not reported): 4 capsules/day for 2 months then 2 capsules/day for 4 months Placebo–same dosing schedule	Lyprinol^®^ group: significant improvement vs. placebo for VAS Pain, GA. NSD in % change in pain killer use in groups (no between group analysis reported)
RCT (*n* = 6) [50]	Intervention–Seatone capsules (dosage not reported) Placebo–dosage not reported	NSD between groups for all outcome measures (VAS Pain, GS, RAI, LUT, TW)
RCT (*n* = 47) [51]	Intervention–Seatone (1050 mg/day) Placebo–dried fish placebo Treatment period–12 weeks	NSD between groups for VAS, GS, RAI, DP, NP, LUT, analgesic intake and GA
RCT (*n* = 35) [52]	Intervention–Seatone 920 mg/day for 6-months Placebo–Placebo capsules for 6 months	NSD between groups for all outcome measures at 0, 3 and 6-month assessments (RAI, GS, LUT and VAS)
RCT (*n* = 38) [17]	Intervention–3000 mg/day whole GLM extract Placebo–3000 mg/day GS p.o. Treatment period = 12 weeks	NSD bacterial growth patterns Both groups showed decreasing trend in *Clostridium* and *Staphylococcus*. Trend of increase growth in *Lactobacillus, Streptococcus* and *Eubacterium* species GLM group: Bifidobacterium increased and Enterococcus & yeast species decreased GS group: decreasing trend in *Bacteroides* and an increase in yeasts and Coliforms species (i.e., *E. coli*) Significant improvement in all OA outcome measures for both groups The GSRS scores indicate gut function significantly improved over treatment period
RCT (*n* = 50) [53]	Intervention–GLM extract capsule (50 mg extract/capsule) + D-002 (50 mg)/day for 6 weeks Placebo–GLM extract capsule (50 mg extract/capsule) + placebo	Both groups: significantly reduced total WOMAC score, pain and physical function WOMAC sub-scores and VAS scores compared to baseline Values achieved in intervention group lower than placebo
RCT (*n* = 50) [24]	Group A = PCSO-524™ (4 capsules/day = 1200 mg/day) Group B = Fish oil (150 mg/capsule) Treatment = 12 weeks	Group A: significant improvement compared to Group B (pain symptoms and QOL) Group B: significantly greater level of physical discomfort
Open clinical trial (*n* = 60) [54]	2 capsules twice a day of Lyprinol^®^ for 8 weeks Parameters analyzed at 4 and 8 weeks	Significant improvement in VAS, LI and physician global assessment in Lyprinol^®^ group No adverse effects reported

RCT: randomised controlled trial, NSD: not significant difference; AI: articular index; VAS: visual analogue scale; FI: functional index; RAI: Ritchie articular index of joint tenderness; LUT: morning joint stiffness or limbering-up time; GS: grip strength; TW: time to walk set distance or maximum walking distance; DP: severity of day-time pain; NP: severity of night-time pain; GA: patient and physician’s global assessment; QOL: quality of life.
